# The Therapeutics of Bright’s Disease Based upon Its Etiology

**Published:** 1907-09

**Authors:** 


### THE THERAPEUTICS OF BRIGHT'S DISEASE BASED UPON ITS ETIOLOGY.

Porter says that under the common term Bright's disease there are three general classes to be considered: First, one in which the pathologic changes are confined largely to the epithelial elements constituting the renal structures, and classed as a parenchymatous lesion. Second, one in which the major lesion is located between the tubules, in the form of a more or less abundant production of newly formed connective tissue substance, and classed as an interstitial lesion. Third, one in which the epithelial and connective tissue elements are more or less equally rendered pathologic and classed as a diffuse lesion. The author differentiates thirteen different renal lesions in a comparative table, showing the urinary changes. In order to intelligently treat Bright's disease it is necessary to have a full understanding of the etiologic factors which the author's article fully embraces. He considers the therapy under two common headings; first, that of prevention, and second, that which is applicable after positive development of the renal lesion. The following are the writer's deductions: 1. All therapeutics to be scientific, must have for the primary object the removal of the causative factors that are producing the pathologic conditions. There are three general subdivisions of the renal lesions classed as "Bright's." 3. Each of these is further subdivisible, both from a pathologic and clinical standpoint. 4. Hemorrhagic nephritis as such does not exist. 5. The so-called hemorrhagic nephritis is an occasional modification of the chronic parenchymatous change. 6 Most renal lesions are non-inflammatory processes. 7. Most renal lesions are due to overwork at a time when nutrition is impaired. 8. The one exception is in condition with the class known as an exudative or inflammatory process. Here all the phenomenon of a true inflammation exists. 9. There are three well defined causative factors which enter into the production of the parenchymatous group of lesions. 10. These three factors, plus five others, enter into the production of the intertitial and diffuse group of lesions. 11. Both the overuse and defective supply of starches, sugars and fats are important factors to be considered in the production, maintenance and cure of these renal lesions. 12. Nervous overstrain is an important factor which often acts indirectly in exciting and maintaining renal affections. 13. Treatment is to be directed both to prevent and to cure. 14. Treatment consists chiefly in the correct apprehensions and removal of the causative factors. 15. The successful management of this class of pathologic lesions calls for an extensive and thorough knowledge of chemicopathology and dietetics. 16. Drugs are not in any sense curative; but when rightly used can be made to produce wonderful results by enabling nature to utilize successfully the natural food substances; thus nature can be made both to remove the cause and repair the damage. 17. These renal lesions when studied purely from the histologic standpoint are incurable. 18. Considered from the physiologic and chemical standpoint, a larger per cent are curable. 19. Viewed from the standard it is difficult to see how surgical interference can, even in a limited degree be made to remove the underlying factors, hence such measures cannot be classed as scientific.—American Medicine, 1907.

